# Data showing effects of resolvin D_5_ on prostaglandin E_2_ mediated inhibition of fMet-Leu-Phe induced activation of the NADPH oxidase in human neutrophils

**DOI:** 10.1016/j.dib.2025.112080

**Published:** 2025-09-19

**Authors:** Wenyan Li, Claes Dahlgren, Huamei Forsman

**Affiliations:** a Department of Respiratory Medicine, Guangzhou Women and Children’s Medical Centre, Guangzhou Medical University; Guangdong Provincial Clinical Research Centre for Child Health, Guangzhou 510623, China; b Department of Rheumatology and Inflammation research, University of Gothenburg, Gothenburg 40530, Sweden; c Department of Clinical Chemistry, Sahlgrenska University Hospital, Gothenburg 41345, Sweden

**Keywords:** G protein coupled receptors, SPNs, EP4, PGE2, FPR1, Allosteric modulation

## Abstract

Regulation of the reactive oxygen species (ROS) producing NADPH oxidase, expressed in neutrophils, is essential for a balance between the proinflammatory antimicrobial host defence and the resolving reactivity that limits tissue destructing inflammatory processes. Peripheral blood neutrophils of healthy adults were isolated from buffy coats obtained from the blood bank at Sahlgrenska University Hospital, using a standard density-gradient centrifugation protocol. The neutrophils were activated by fMet-Leu-Phe (fMLF) a peptide recognized by formyl peptide receptor 1 (FPR1). Signals generated downstream of the agonist occupied FPR1 activate the NADPH oxidase in the neutrophil plasma membrane. The release of ROS by fMLF activated neutrophils was measured in real time using a very sensitive isoluminol-amplified chemiluminescence system, expressed in light units (Mega counts per minute; Mcpm), and the peak value levels of the responses were determined. The activation signals generated by FPR1 were inhibited (reduced peak values) by the agonist occupied EP_4_, a neutrophil receptor for prostaglandin E_2_ (PGE_2_). The inhibitory effect of PGE_2_ was expected to be increased (positively modulated) by Resolvin D_5_ (RvD_5_), generated from the omega-3 fatty acid docosahexaenoic acid and a member of the group of specialized pro-resolving lipids. The dataset presented in the article includes raw data on the effects of RvD_5_ on the inhibitory activity of PGE_2_. The reuse of data indicating a lack of inhibition of a negative allosteric modulator on ROS production in neutrophils is high and multifaceted across several different research domains such as drug development (drug effects lies elsewhere or should be re-evaluated) and immunological research (neutrophil studies and animal models and as reference for testing the robustness or specificity of other ROS-detecting assays or biosensors).

Specifications Table

**Table:** 

Subject	
Specific subject area	G protein coupled receptor (GPCR) signaling, ResolvinD_5_ (RvD_5_), prostaglandin E_2_ (PGE_2_), prostaglandin E_2_ receptor (EP_4_), formyl peptide receptor 1 (FPR1), specialized pro-resolving mediators (SPMs), human neutrophils, inflammation
Type of data	Table, Graph, Raw, Analysed, Processed.
Data collection	Human blood neutrophils isolated from buffy coats were incubated with PGE_2_ in the presence or absence of the allosteric modulator RvD_5_. The neutrophils were then activated by the FPR1 agonist fMLF and receptor downstream function was monitored as production of superoxide anions (O_2_^−^) through the neutrophil NADPH-oxidase. Production of O_2_^−^ was measured by an isoluminol-enhanced chemiluminescence system which records light emission continuously over time using a chemoluminometer (Biolumat LB 9505; Berthold Co, Wildbad, Germany). The light emission/O_2_^−^ production, expressed in Mcpm, was analysed.
Data source location	Department of Rheumatology and Inflammation Research, Institute of Medicine, Sahlgrenska Academy, Gothenburg University, Gothenburg, Sweden
Data accessibility	Repository name: Mendeley Data identification number: 10.17632/mc8nwzvsk2.1 Direct URL to data: https://data.mendeley.com/datasets/mc8nwzvsk2/1
Related research article	None

## 1 Value of the Data

• This dataset is the first to show the effect of RvD_5_ on PGE_2_ mediated inhibition of FPR1 mediated activation of the neutrophils NADPH-oxidase, an electron transporting enzyme system that is essential for microbial killing and the regulation of inflammatory processes.
• These data expand the current knowledge about allosteric modulation and GPCR signalling which may benefit future research focused on the role of neutrophil GPCRs as regulators of inflammation.
• The findings add value to the characterization of SPMs as modulators of inflammatory reactions. These data are relevant for researchers studying innate immune systems and inflammatory processes.

## 2 Background

Human neutrophils are equipped with a multi-component electron transporting NADPH oxidase consisting of membrane bound as well as cytosolic components. When assembled in the plasma membrane the oxidase produces O_2_^−^ that is released extracellularly [1]. Neutrophils activated by the FPR1 (a Gα_i_ coupled member of the G protein coupled receptor family; GPCRs) specific agonist fMLF produce O_2_^−^[2]. This production is inhibited by signals generated by other GPCRs that couple to a Gα_s_ coupled G protein [3]. PGE_2_ (agonist for the Gα_s_ coupled EP_4_ receptor) is one of the GPCRs mediates inhibition on the neutrophil release of O_2_^−^when activated by fMLF. RvD_5_, which is a specialized pro-resolving lipid derived from the omega-3 fatty acid docosahexaenoic acid, was anticipated to enhance the inhibitory effect of PGE₂ through positive modulation. [4]. The main objective of the experiments performed was to explore how the allosteric modulator RvD_5_ affects the inhibitory activity of PGE₂, when neutrophils were activated by the FPR1 agonist fMLF.

## 3 Data Description

This article describes the dataset of the linked repository of raw data of an isoluminol-amplified chemiluminescence system, which measured ROS production by neutrophil NADPH oxidase in real time along with the annotated experimental protocol.

**Fig. 1:**
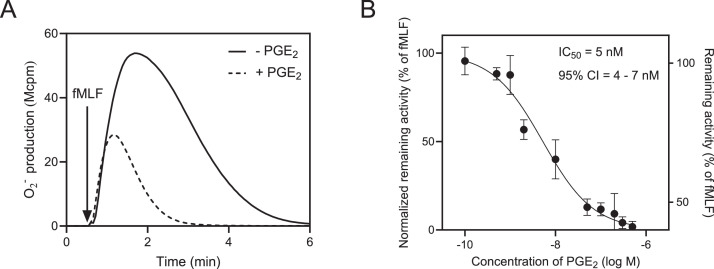
PGE2 affects NADPH oxidase activity induced by fMLF in human neutrophils A) Neutrophils were incubated with and without PGE_2_ (500 nM) for 5 min at 37 °C and then activated by fMLF (100 nM, final concentration). The NADPH oxidase produced superoxide anions (O_2_^−^) were measured over time. The addition of the activating FPR1 agonist is indicated by an arrow. One representative experiment out of 5 individual experiments is shown. **B)** Neutrophils were incubated with different concentrations of PGE_2_ for 5 min at 37 °C and then activated by fMLF (100 nM, final concentration). The neutrophil NADPH oxidase activity was measured as O_2_^−^ production and the peak activity value was used to determine the PGE_2_ mediated inhibition. The half-maximal inhibitory concentration (IC_50_) of PGE_2_ was calculated (95 % confidence interval [CI]; mean ± SD, *n* = 3). The right vertical axis represents the peak value of the actual remaining activity as a percentage of the peak value induced by fMLF without PGE₂. The vertical axis to the left represents normalized inhibition (remaining activity) values, where no inhibition (100 %) corresponds to the peak fMLF response without any PGE_2_ and full inhibition (0 %) corresponds to the peak fMLF response in the presence of the PGE_2_ concentration (500 nM) giving a maximal inhibitory activity. The vertical axis to the right represents real inhibition values, where no inhibition (100 %) corresponds to the peak fMLF response without any PGE_2_ and maximal inhibition (0 %) corresponds to the peak fMLF response in the presence of the PGE_2_ concentration of 500 nM.

Data describes the effect of RvD_5_ on the inhibition mediated by PGE_2_ on the neutrophil release of O_2_^−^when activated by fMLF. Activation of the NADPH oxidase induced by fMLF (100 nM final concentration) in the absence and presence of PGE_2_ (500 nM final concentration is shown (Fig. 1A). Also, data on the effect of different concentrations of PGE_2_ on the fMLF is provided; the remaining activity with high concentrations (above 300 nM) of PGE_2_ reached ≈40 % and the IC_50_ value was 5 nM (Fig. 1B).

**Fig. 2:**
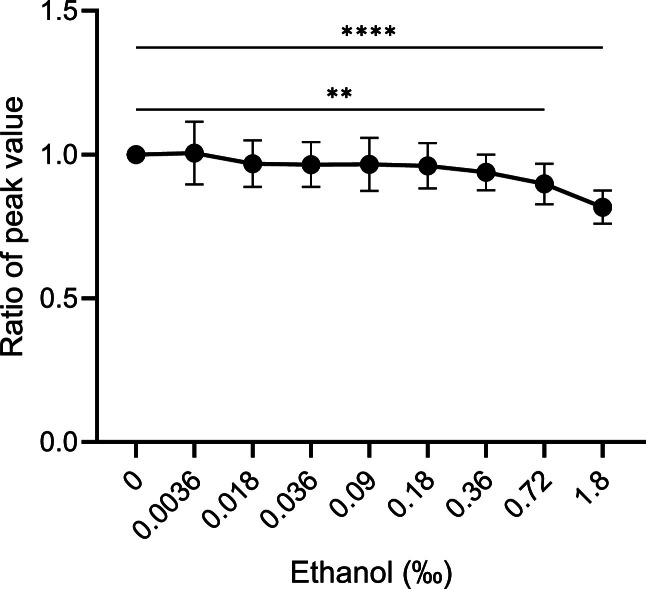
Inhibitory effect of ethanol on fMLF-induced NADPH oxidase activity in human neutrophils. Neutrophils were pre-treated with PGE_2_ (0 nM, 1 nM, and 300 nM, respectively) in presence of different concentrations of ethanol as indicated for 5 min at 37 °C. The neutrophils were then activated by fMLF (100 nM). The neutrophil NADPH oxidase-derived O_2_^−^ was measured over time. The data on the peak value ratios calculated are shown in Table 1 and used to show the effects on O_2_^−^ production of different concentrations of ethanol. Statistically significant differences were evaluated by ordinary one-way ANOVA with Dunnett's multiple comparisons test (**p < 0.01, ****p < 0.0001).

The allosteric modulator RvD_5_ was dissolved in ethanol. Data describes the effect of different concentrations of the RvD_5_ solvent (ethanol from 0 to 1.8 ‰) on the activation of the neutrophil NADPH oxidase induced by fMLF (100 nM final concentration) alone and in the presence of PGE_2_ (1 and 300 nM respectively; Table 1). Also, data showing effects of different concentrations of ethanol when the data obtained with different concentrations of PGE_2_ were combined are provided; the fMLF induced response was decreased with the two highest concentrations of ethanol (the ratio value with 0.72 ‰ was 0.90 and with 1.8 ‰ was 0.82; Fig. 2).

**Table 1:** Effect of ethanol on NADPH oxidase activity induced by fMLF with PGE_2_ pretreatment (ratio of peak values; mean ± SD).

PGE_2_ (nM)	Ethanol (‰)								
	0	0.0036	0.018	0.036	0.09	0.18	0.36	0.72	1.8
0	1	0.93 ± 0.16	0.94 ± 0.07	0.92 ± 0.05	0.93 ± 0.02	0.94 ± 0.13	0.92 ± 0.05	0.88 ± 0.05	0.81 ± 0.06^⁎⁎^
1	1	1.02 ± 0.02	0.94 ± 0.03	0.99 ± 0.06	0.95 ± 0.05	0.98 ± 0.08	0.94 ± 0.07	0.94 ± 0.08	0.81 ± 0.07^⁎⁎⁎^
300	1	1.06 ± 0.09	1.02 ± 0.12	0.98 ± 0.12	1.02 ± 0.16	0.96 ± 0.04	0.96 ± 0.08	0.88 ± 0.08	0.83 ± 0.06*
Total^a^	1	1.01 ± 0.11	0.97 ± 0.08	0.97 ± 0.08	0.97 ± 0.09	0.96 ± 0.08	0.94 ± 0.06	0.90 ± 0.07^⁎⁎^	0.82 ± 0.06^⁎⁎⁎⁎^

The peak value ratio is calculated as:

$Ratio=\frac{\text{Peak}\;\text{value}\;\text{of}\;O2^{-}\;\text{production}\;\text{induced}\;\text{by}\;\text{fMLF}\;\left(100\;\text{nM}\right)\;\text{with}\;\text{ethanol}\;+\;\text{PGE}2\;\text{pretreatment}}{\text{Peak}\;\text{value}\;\text{of}\;O2^{-}\;\text{production}\;\text{induced}\;\text{by}\;\text{fMLF}\;\left(100\;\text{nM}\right)\;\text{with}\;\text{PGE}2\;\text{pretreatment}}.$

^a^ The total is calculated by all peak value ratio of O_2_^−^production induced by fMLF (100 nM) pre-treated by three concentrations of PGE_2_ (0, 1, and 300 nM) with ethanol at varying concentrations (‰).

Statistically significant differences were evaluated by ordinary one-way ANOVA with Dunnett's multiple comparisons test (*p < 0.05, **p < 0.01, ***p < 0.001, ****p < 0.0001). 3–5 individual experiments for each concentration of PGE_2_ (0, 1, and 300 nM) with different concentrations of ethanol (‰).

**Fig. 3:**
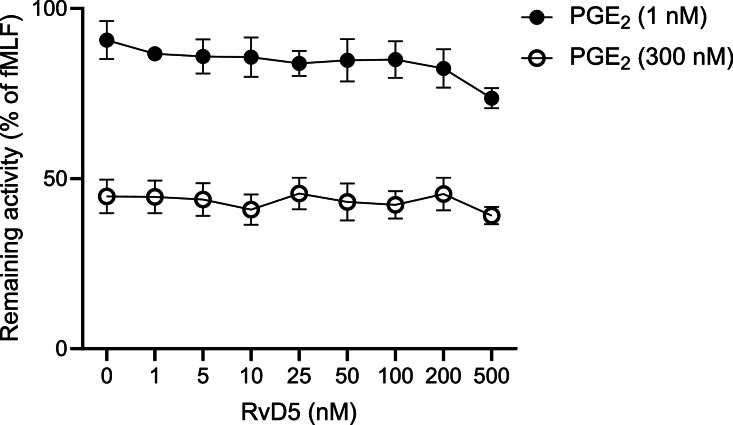
Effects of RvD5 on the inhibition mediated by PGE₂ on fMLF-induced NADPH oxidase activity in human neutrophils. Neutrophils were incubated with PGE_2_ (1 nM; ● and 300 nM; ○, respectively) in the absence (no RvD_5_) or presence of RvD5 5 min at 37 °C. The cells were then activated by fMLF (100 nM) and the NADPH oxidase-derived oxygen O_2_^−^ production was measured over time. The peak value of the activity in the prence of RvD_5_, expressed in percentage of the peak value of the activity induced by induced by fMLF without RvD5 pretreated is shown (mean ± SD, *n* = 4). Statistically significant differences were evaluated by RM one-way ANOVA with Dunnett's multiple comparisons test (*p < 0.05).

Data are shown that describe the change in the activity of the neutrophil NADPH oxidase induced by fMLF (100 nM final concentration) in the presence of PGE_2_ (1 and 300 nM respectively) when combined with different concentrations of RvD_5_ (from 0 to 500 nM; Fig. 3).

## 4 Experimental Design, Materials and Methods

### 4.1 Separation of human peripheral blood neutrophils

Human peripheral blood neutrophils were isolated from healthy donors’ buffy coats using dextran sedimentation, Ficoll-Paque gradient centrifugation and removal of residual erythrocytes by hypotonic lysis, as described previously [5,6]. The cells were diluted to 1 × 10^6^/mL in Krebs-Ringer phosphate buffer (KRG) containing glucose (10 mM), Mg^2+^(1.5 mM), and Ca^2+^ (1 mM), and kept to room temperature (RT) for 1 hour, mixed every 15 min, and then placed on ice until use.

### 4.2 Measurement of the NADPH oxidase activity

Extracellular O_2_^−^ production induced by GPCRs agonists was measured by an isoluminol-enhanced chemiluminescence system, in which isoluminol reacts with superoxide in the presence of horseradish peroxidase (HRP) to generate a light signal, which is continuously monitored by a luminometer and provides a real-time measure of extracellular ROS production [7]. The release of O_2_^−^ was measured in the six channel Biolumat LB 9505 (Berthold Co, Wildbad, Germany). Disposable 4 mL polypropylene tubes with a 0.9 mL reaction mixture were used. Neutrophils (1 × 10^5^), HRP (4 U/mL), isoluminol (10 µg/mL) and agonist/inhibitor (KRG for control) were incubated (five min at 37 °C) before stimulation with 0.1 mL of the FPR1 agonist fMLF and the production of O_2_^−^was measured as light emission over time. Data are shown with a representative chemiluminescence kinetics, abscissa time (min) and ordinate chemiluminescence arbitrary units (Mega counts per minute, Mcpm).

### 4.3 Materials

Prostaglandin E_2_ (PGE_2_) was obtained from Bio-Techne/Tocris (Dublin, Ireland, Cat. No. 2296) and Resolvin D_5_ (RvD_5_) dissolved in ethanol was obtained from Cayman Chemicals (Ann Arbor, Michigan, USA, Cat. No. 154–10,007,280). The peptide ligand fMLF, specifically recognized by formylpeptide receptor 1 (FPR1; [8]) purchased from Sigma-Aldrich (St. Louis, MO, USA, Cat. No. F3506). Ethanol absolute purchased from VWR BDH™ (Avantor, Radnor, PA, USA, Cat. No. 20,821.296). Krebs-Ringer phosphate buffer supplemented with 120 mM NaCl, 4.9 mM KCl, 1.5 mM MgSO_4_, 1.7 mM KH_2_PO_4_, 8.3 mM Na_2_HPO_4_, 10 mM glucose, and 1 mM CaCl_2_ in dH_2_O (KRG; pH 7.3) was made in house. Dextran T500 was obtained from Pharmacosmos (Holbæk, Denmark, Cat. No. 5510 0500–9007). Cytiva Ficoll-Paque™ Plus Medium was obtained from Fischer Scientific (Gothenburg, Sweden, Cat. No. 17,144,003). Horseradish peroxidase (HRP) was obtained from Roche (Mannheim, Germany, Cat. No. 10,108,090,001). Isoluminol was from Sigma-Aldrich (St. Louis, MO, USA, Cat. No. A8264).

### 4.4 Data analysis

Data analysis was performed with Graph Pad Prism 10 version 10.1.0 (GraphPad Software, La Jolla, CA, USA). Data were analyzed with a paired Student´s *t*-test or a one-way ANOVA followed by Dunnet's multiple comparison test; details are stated in the respective figure legends. Statistically significant differences are indicated by *p ≤ 0.05. **p ≤ 0.01. Each independent experiment was performed as biological replicates with neutrophils isolated from individual blood donors.

## Limitations

In this study, we evaluated only the production of extracellular O₂⁻ by neutrophils as a representative functional response. Other known inhibitory effects of PGE₂ on human neutrophil functions—such as leukotriene B_4_ (LTB₄) biosynthesis, granule secretion and cell migration—were not examined. Therefore, our findings offer a limited perspective on the influence of RvD_5_ on PGE₂ activity in human neutrophils. In addition, the experiments were conducted under defined in vitro conditions with isolated neutrophils, which may not fully reflect the complexity of the in vivo environment.

## Ethics Statement

Buffy coat blood samples were obtained from healthy adults from the blood bank at Sahlgrenska University Hospital. Ethics approval was not required because the buffy coats were provided anonymously and could not be traced back to a specific individual. This is in accordance with the Swedish Ethical Review Act, which refers to research involving humans, Swedish legislation section code 4§ 3p SFS 2003:460 (Lag om etikprövning av forskning som avser människor).

## CRediT Author Statement

**Wenyan Li**: Methodology, Formal analysis, Data curation, Investigation, Visualization, Writing – Original Draft. **Claes Dahlgren**: Conceptualization, Methodology, Validation, Writing – Original Draft, Project administration, Supervision. **Huamei Forsman**: Validation, Resources, Writing – Review and Editing, Funding acquisition, Supervision.

## Data Availability

Mendeley DataResolvin D5 on prostaglandin E2 mediated inhibition of fMLF induced activation of the neutrophil NADPH (Original data)

## References

[1] Dahlgren C., Karlsson A., Bylund J. (2019). Intracellular neutrophil oxidants: from laboratory curiosity to clinical reality. J. Immunol..

[2] Dahlgren C., Forsman H., Sundqvist M., Bjorkman L., Martensson J. (2024). Signaling by neutrophil G protein-coupled receptors that regulate the release of superoxide anions. J. Leukoc. Biol..

[3] Betten A., Dahlgren C., Hermodsson S., Hellstrand K. (2003). Histamine inhibits neutrophil NADPH oxidase activity triggered by the lipoxin A4 receptor-specific peptide agonist Trp-Lys-Tyr-met-val-met. Scand. J. Immunol..

[4] Alnouri M.W., Roquid K.A., Bonnavion R., Cho H., Heering J., Kwon J., Jager Y., Wang S., Gunther S., Wettschureck N., Geisslinger G., Gurke R., Muller C.E., Proschak E., Offermanns S. (2024). SPMs exert anti-inflammatory and pro-resolving effects through positive allosteric modulation of the prostaglandin EP4 receptor. Proc. Natl. Acad. Sci. U S A.

[5] Boyum A. (1984). Separation of lymphocytes, granulocytes, and monocytes from human blood using iodinated density gradient media. Methods Enzymol..

[6] Forsman H., Onnheim K., Andreasson E., Dahlgren C. (2011). What formyl peptide receptors, if any, are triggered by compound 43 and lipoxin A4?. Scand. J. Immunol..

[7] Dahlgren C., Bjornsdottir H., Sundqvist M., Christenson K., Bylund J. (2020). Measurement of Respiratory burst products, released or retained, during activation of professional phagocytes. Methods Mol. Biol..

[8] Dahlgren C., Lind S., Martensson J., Bjorkman L., Wu Y., Sundqvist M., Forsman H. (2023). G protein coupled pattern recognition receptors expressed in neutrophils: recognition, activation/modulation, signaling and receptor regulated functions. Immunol. Rev..

